# Event-Related Potentials Elicited by Face and Face Pareidolia in Parkinson's Disease

**DOI:** 10.1155/2020/3107185

**Published:** 2020-03-31

**Authors:** Gulsum Akdeniz, Gonul Vural, Sadiye Gumusyayla, Hesna Bektas, Orhan Deniz

**Affiliations:** ^1^Department of Biophysics, Department of Neuroscience, Electroneurophysiology Lab, Ankara Yildirim Beyazit University, School of Medicine, Yenimahalle Training and Research Hospital, Ankara, Turkey; ^2^Department of Neurology, School of Medicine, Ankara Yildirim Beyazit University, Ankara, Turkey

## Abstract

**Background:**

Parkinson's disease is associated with impaired ability to recognize emotional facial expressions. In addition to a visual processing disorder, a visual recognition disorder may be involved in these patients. Pareidolia is a type of complex visual illusion that permits the interpretation of a vague stimulus as something known to the observer. Parkinson's patients experience pareidolic illusions. N170 and N250 waveforms are two event-related potentials (ERPs) involved in emotional facial expression recognition.

**Objective:**

In this study, we investigated how Parkinson's patients process face and face-pareidolia stimuli at the neural level using N170, vertex positive potential (VPP), and N250 components of event-related potentials.

**Methods:**

To examine the response of face and face-pareidolia processing in Parkinson's patients, we measured the N170, VPP, and N250 components of the event-related brain potentials in a group of 21 participants with Parkinson's disease and 26 control participants.

**Results:**

We found that the latencies of N170 and VPP responses to both face and face-pareidolia stimuli were increased along with their amplitudes, and the amplitude of N250 responses decreased in Parkinson's patients compared to the control group. In both control and Parkinson's patients, face stimuli generated greater ERP amplitude and shorter latency in responses than did face-pareidolia stimuli.

**Conclusion:**

The results of our study showed that ERPs associated with face and also face-pareidolia stimuli processing are changed in early-stage neurophysiological activity in the temporoparietal cortex of Parkinson's patients.

## 1. Introduction

Parkinson's disease (PD) is more than a motor system movement. Cognitive and sensory processing may, both, also be impaired, and even such nonmotor symptoms can often be more stringent [1]. PD is associated with impaired ability to process facial expressions [2]. In addition to visual processing disorder, visual misperception may be involved in these patients.

The electroencephalogram (EEG) registers brain electrical activity research on Parkinson's disease is of low cost and represents no risk to the patient. Most of these studies are focused on resting EEG, and less studies on event-related potentials (ERPs). N170 and N250 waveforms are two ERPs in relation to face processing. N170 is an evoked potential that can be stimulated by presentation of various facial expression pictures or line drawings, regardless of their positioning (e.g., vertical and upside down) or accuracy (e.g., a distorted image). N170 was recorded approximately 170 milliseconds (ms) after the stimulus. That is, N170 represents the neural mechanism that permits detection of facial expression, and this waveform reflects activity of the occipitotemporal areas of the brain [3]. The N250 ERP peaks approximately 250 ms after the stimulus and is recorded from the frontocentral area. Although N250 is triggered by same stimulus as N170, N250 is modified by the affective content of the face [4], its familiarity [5], and repetitive stimuli [6]. N250 is very sensitive for facial expression identity [7]. The vertex positive potential (VPP), such as N170, is a face-selective ERP recorded from the frontal lobe of the brain [8]. VPP amplitude and latency are correlated with N170 [9].

The ERPs displays abnormally lower frequencies in some patients with Parkinson's disease (PD), but only rarely this issue has been studied with visual paradigms [10–12]. ERP studies of the P3 component of a visual oddball task are delayed in PD in patients with a history of visual hallucinations [13]. In association with visual illusions, delay in evoked potential latency was also reported in an immersive virtual reality environment following short-term PD patient's medication deprivation [14]. Additionally, previous neuropsychological studies have demonstrated that a subset of PD patients without visual hallucinations exhibited illusory misperceptions of nonexistent visual objects [15, 16]. This uncover with such neuropsychological tests may represent a predisposition to visual illusory in PD patients.

Pareidolia, are complex visual hallucination-like illusions involving ambiguous forms that is the interpretation of previously unseen and unrelated objects as familiar due to previous learning [17]. Patients with PD without dementia have been reported to be present in about visual hallucinations 10% [18–20] and visual illusion in 6–19% [19, 20]. The use of pareidolia paradigm with ERP to measure clinically important phenomena is in its infancy. Although pareidolia is a normal perception in healthy individuals, it is increased in frequency in patients with Parkinson's disease [21]. A pareidolia test has been developed for visual hallucinations in dementia with Lewy bodies who experience visual hallucinations that may also have a role in the assessment in PD psychosis [22]. The positron emission tomography study with pareidolia has revealed that pareidolia could represent subclinical hallucinations for Parkinson's disease without dementia.

There have been a few experimental studies that perform the underlying mechanisms of illusion face, but PD is still remaining unclear. Based on the information in the literature so far, we assume that face pareidolia would be observed in PD patients without dementia and that both real face and face pareidolia would be associated with activity in specific regions of the brain, especially temporoparietal cortices. In this study, we have tested this hypothesis on how PD patients process face and face-pareidolia stimuli using N170, VPP, and N250 components of ERPs.

## 2. Methods

### 2.1. Participants

The study was approved by the Ethics Committee of Ankara Yıldırım Beyazıt University. Twenty-one participants with Parkinson's disease without dementia (15 males) had been referred to the movement disorders clinic of the Department of Neurology of the Ankara Atatürk Training and Research Hospital. The diagnosis of PD was based on the criteria of the “United Kingdom Parkinson's Disease Society Brain Bank” [23]. Detailed clinical assessment of all patients was performed by a neurologist. The Unified Parkinson's Disease Rating Scale (UPDRS) [24] and Mini-Mental Test for General Cognitive Assessment (MMSE) [25] were used in order to determine the clinical features of PD; and the Hoehn–Yahr scale [26] was used to determine the disease stage. Patients with a history of other neurological, psychiatric, or ocular disease, or the presence of dementia, according to the Diagnostic and Statistical Manual of Mental Disorders, were excluded from the study. Twenty-six healthy volunteers (14 males) were included as controls. Healthy volunteers reported no history of neurological or psychiatric disorders. All subjects gave their consent in writing and had normal or corrected to normal vision.

### 2.2. Data Recording, Stimuli, and Data Processing

A 32-channel EEG was recorded with an ActiCHamp (Brain Products GmbH, Gilching, Germany). The sampling rate was 256 Hz, and the reference electrode was Cz, which was re-referenced to average. Impedances were kept below 10 kΩ. Artifact-free segments were visually selected. EEGs were filtered (bandpass: 0.16–100 Hz, notch: 50 Hz). Artifacts such as muscle activity or eye blinks were detected and omitted by the Brain Vision Analyzer 2 (Brain Products, Munich, Germany). Channels with bad activations were interpolated (by the spherical spline method). The motor movement during the action of pushing the button was constant since it was the entire experiment. An automated peak detection analysis was performed for the N170 (160–190 ms) time interval at P7, TP7, P8, and TP8 electrodes, the VPP (160–190 ms) time interval at F3, FC3, C3, F4, FC4, and C4 electrodes, and the N250 (200–250 ms) time interval at Fp1, F3, Fp2, and F4 electrodes.

**Figure 1:**
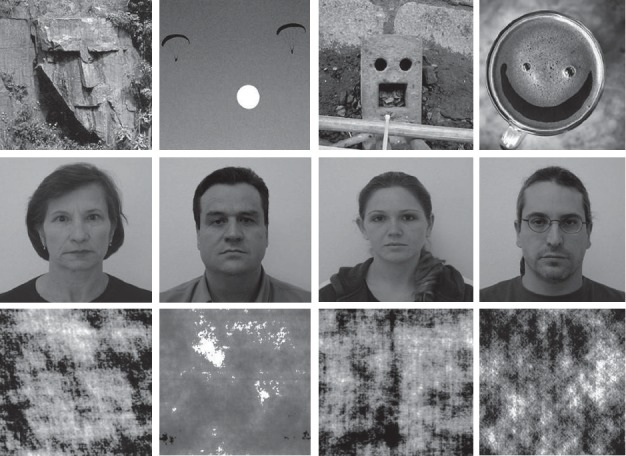
Illustration of example of face pareidolia (first row), face (second row), and scrambled face (third row) images.

Photographs of faces were obtained from the Centro Universitário da FEI. Face-pareidolia images were obtained from the Internet by searching for pareidolia. Face-scrambled and face-pareidolia-scrambled images were created in the SHINE Toolbox in MATLAB (MathWorks, Inc., Natick, MA, USA) from the face images and face-pareidolia images, respectively, to use as the untarget image (Figure 1).

The stimulus was presented on a 19-inch LED computer monitor in an isolated room. A small, red circle was used as a fixation point. There were 2 trials of 80 stimuli; the first part consisted of 40 face and 40 face-scrambled images, and the second part consisted of 40 face pareidolia and 40 pareidolia-scrambled images (Figure 1). Participants were asked to press a button if they perceived any real face or face-like images.

### 2.3. Statistical Analysis

The mean peak amplitudes and latencies of N170, VPP, and N250 were calculated for each participant. SPSS 12.0 software was employed for statistical analysis (SPSS, Chicago, IL). Statistical analysis was performed with repeated measures of the analysis of variance (ANOVA) included in SPSS software. In the first statistical session (behavioral data), we tested the hypothesis of higher accuracy (i.e., percentage of correct responses) and shorter reaction time in behavioral responses of the PD patients compared with the healthy controls in the face condition and the face-pareidolia condition (*p* < 0.05). This hypothesis was evaluated by an ANOVA having the response accuracy as a dependent variable and group (PD patients and healthy controls; independent variable) and condition (face and face pareidolia) as factors. Similarly, another ANOVA used the reaction time as a dependent variable and group (PD patients and healthy controls; independent variable) and condition (face and face pareidolia) as factors. In the second statistical session (EEG data), we tested the hypothesis of mean differences in the components of ERPs between the groups of PD patients and healthy controls in the face and the face-pareidolia condition (*p* < 0.05). The ERP data were subjected to multifactorial repeated measures ANOVA. The between-group factor was group (PD patients and healthy controls) and the within-group factors were condition (face and face pareidolia) and electrode (the ERP of interest: P7, TP7, P8, and TP8 for N170 amplitude and latency; F3, FC3, C3, F4, FC4, and C4 for VPP amplitude and latency; Fp1, F3, Fp2, and F4 for N250 amplitude and latency). The results of this statistical analysis were controlled by the Grubbs test (*p* < 0.001) for the presence of outliers.

## 3. Results

PD (15 males; mean age: 57.62 ± 8.04 years) patients and 26 aged and sex-matched healthy participants (14 males; mean age: 56.22 ± 7.74) were involved in the study. The average PD duration was 2.2 ± 1.2 years (min. 1 yr. and max 5 yrs.). According to the Hoehn and Yahr scales, 15 patients were evaluated as at stage 1 (71.4%), 2 patients were at stage 1.5 (9.5%), and 4 patients were at stage 2 (19%). The demographic characteristics are presented in Table 1.

In the PD patients, the mean accuracy of behavioral responses was 98% (±1.2 SE = standard error) in the face condition and 95% (±1.4 SE) in the face-pareidolia condition. In the healthy controls, the mean accuracy was 99% (±0.6 SE) in the face condition and 98% (±0.8 SE) in the face-pareidolia condition. In the PD patients, the mean reaction time of behavioral responses was 484 ms (±23 SE) in the face condition and 602 ms (±24 SE) in the face-pareidolia condition. In the control healthy, the mean reaction time was of 475 ms (±62 SE) in the face condition and 594 ms (±72 SE) in the face-pareidolia condition. The ANOVA showed no statistically significant differences between the two groups or between the conditions (*p* > 0.05).

Table 2 presents the results of the average latencies, amplitudes, and SE of N170, N250, and VPP components.

**Figure 2:**
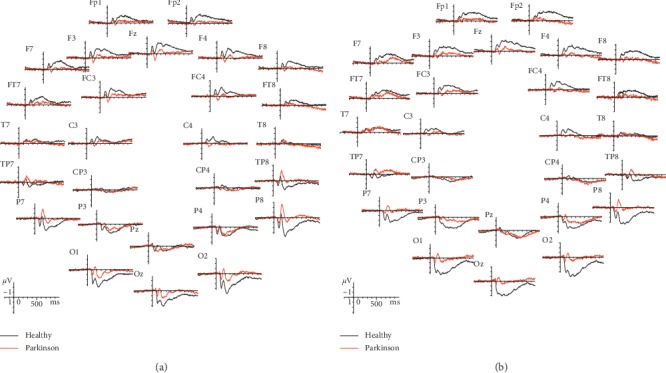
Grand average ERP waveforms recorded overall scalp sites as a function of stimulus class: (a) face grand average (b) face pareidolia grand average.

Grand average waveform to face and face pareidolia is displayed in Figure 2.

### 3.1. N170 (160–190 ms)

**Figure 3:**
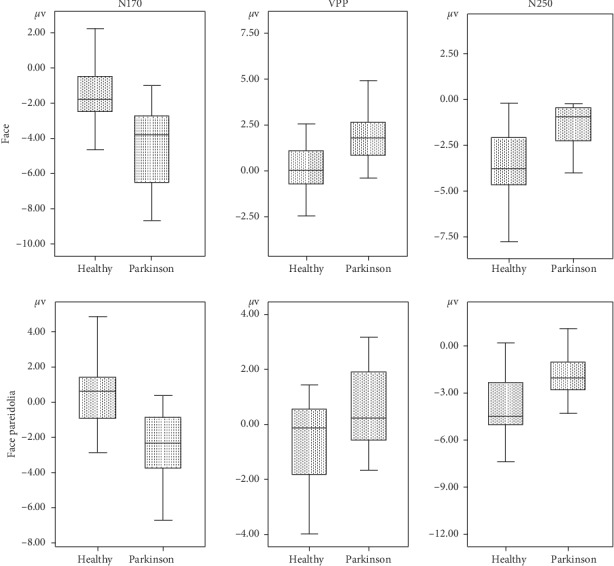
Mean N170, VPP, and N250 amplitude graphics of face and face-pareidolia stimuli between healthy and Parkinson groups.

**Figure 4:**
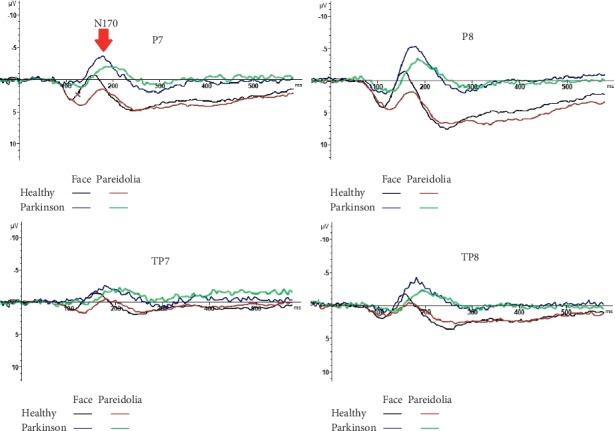
N170 responses to face and face-pareidolia stimuli in healthy and control groups.

The ANOVA carried out on N170 amplitude at occipito/temporal sites (P7, TP7, P8, and TP8) showed a significant difference between groups for face pareidolia (*F*(1,43) = 22.23, *P* < 0.001). The N170 latency elicited by face was significantly earlier in healthy controls compared with Parkinson patients (*F*(1,44) = 18.33, *P* < 0.001; Figure 3). Also, the N170 was significantly earlier in healthy controls than Parkinson patients (*F*(1,43) = 8.64, *P* < 0.05; Figure 4) for face pareidolia. There was a significant difference between stimulus types in healthy controls; faces evoked earlier N170 responses than face pareidolias (*F*(1,50) = 23.88, *P* < 0.001; Figure 4). Also, there was a significantly earlier N170 response for face than face pareidolia in Parkinson patients (*F*(1,37) = 4.82, *P* < 0.05).

### 3.2. VPP (160–190 ms)

**Figure 5:**
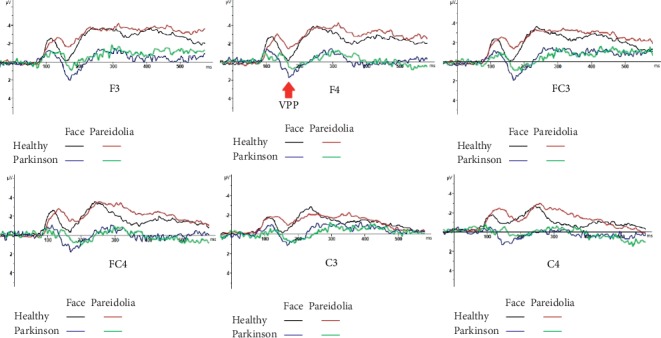
VPP responses to face and face-pareidolia stimuli in healthy and control groups.

The ANOVA comparisons provided evidence of a significantly greater VPP response in Parkinson patients than in healthy controls for both face and face-pareidolia stimuli (*F*(1,44) = 21.37, *P* < 0.001; *F*(1,36) = 4.81, *P* < 0.05, respectively). Also, VPP responses were significantly earlier in healthy controls than in Parkinson patients for both face and face pareidolia (*F*(1,39) = 12.88, *P* < 0.005; *F*(1,43) = 6.26, *P* < 0.05, respectively; Figure 5). The VPP amplitude was significantly greater for face than face pareidolia in Parkinson patients (*F*(1,34) = 9.34, *P* < 0.05; Figure 3).

### 3.3. N250 (200–250 ms)

**Figure 6:**
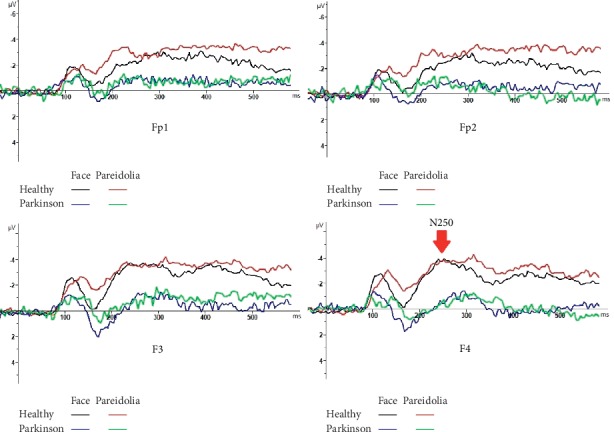
N250 responses to face and face-pareidolia stimuli in healthy and control groups.

The ANOVA showed that the analysis of N250 latency yielded a significant difference between groups for the face; the amplitude of N250 was larger in healthy controls than in Parkinson patients for face (*F*(1,43) = 17.81, *P* < 0.001; Figure 3). The N250 amplitude was also significantly larger in healthy controls than in Parkinson patients for face pareidolia (*F*(1,43) = 8.37, *P* < 0.05; Figure 6).

## 4. Discussion

To our knowledge, ours is the first study to specifically explore the brain areas activated during face and face-pareidolia processing the PD population using ERP. In this study, we examined N170, VPP, and N250 responses to face and face pareidolia in early-stage Parkinson's patients. We found that the latencies of N170 and VPP responses to both face and face-pareidolia stimuli were delayed, and their amplitudes increased. The amplitudes of N250 responses decreased in Parkinson's patients, compared with the control group. In both control and Parkinson's patients, face stimuli revealed greater amplitude and shorter latency responses than face-pareidolia stimuli. Our findings have shown that face pareidolia like face was associated with early neurophysiological activity in the temporoparietal cortex.

ERPs provide the tracking neural correlates of face perception and face recognition [26]. The N170 waveform is documented with higher amplitude in facial stimuli compared with nonfacial stimuli [27]. The neural origin of N170 is from the superior temporal sulcus and fusiform gyrus [28]. An inverted facial expression stimulus elicits both delayed latency and larger amplitude in N170 response. This N170 of amplitude increase and latency delay reflects the deterioration of the holistic/configurational processing of the face [29]. N170 amplitude is also greater in response to emotional faces than to a neutral face [30]. Although the N170 and VPP components are associated with structural face processing, their amplitudes are modulated by facial expression [31]. In our study, we used neutral face and face pareidolia. This was the case in both Parkinson's patients and the control volunteers. The responses obtained in face-pareidolia stimuli were smaller and later in latency than those obtained from face stimuli. Therefore, the face-pareidolia stimuli did not stimulate facial processing networks as significantly as face stimuli. In contrast, both face stimuli and face-pareidolia processing changed in patients with PD, compared with the controls. Neural activity in the visual cortex and the dopaminergic modulation might influence visual cortical processing [32] by long-range interactions originating in the frontal cortex. Thus, we postulate that the evoked larger N170 response in PD patients during face-pareidolia perception may result from altered interaction between top-down and bottom-up brain region modulation from higher frontal cortical areas due to the damage of dopaminergic pathways in the disease. Our findings support the results of Akdeniz's and colleagues' study which demonstrated that fMRI scans were performed on 20 healthy subjects under real-face and face-pareidolia conditions [17]. They found that face pareidolia requires interaction between top-down and bottom-up brain regions including the fusiform face area and frontal and occipitotemporal areas.

There are many studies showing impaired ability in facial processing in patients with PD. Alonso-Recio et al. [33] found that configural processing of the face in Parkinson's patients was not universally impaired, but configural perception of faces does not seem to be globally impaired in PD. However, this ability is selectively altered when the categorization of emotional faces is required as Ariatti et al. [34] suggested that the pattern of disorder in face expression processing in Parkinson's patients depends on the regional distribution of the neuropathology of the disease. Clark et al. [35] showed that there is a specific impairment in the recognition of emotional facial expressions in patients with PD. In this context, although there have been a number of studies investigating emotional facial expression, the mechanisms that may contribute to facial expression processing deficits are not clear [33–37]. One of the main findings of our study was that VPP amplitudes were higher in PD patients than in healthy subjects for both faces and face pareidolias. We speculated that this is related to increased neurodegeneration. In Parkinson's patients, stimulation with emotional facial expression compared to neutral expression is associated with decreased occipital negativity and is further associated with decreased dopaminergic activity in related cortical-subcortical sites [2]. In our results, we obtained decreased activity response to both face and face-pareidolia stimuli in the occipital region and our findings were consistent with the literature.

A functional magnetic resonance imaging (fMRI) study showed decreased activation at the visual motion area (V5), the fusiform gyrus, and the right superior temporal sulcus, in Parkinson's patients. This decreased activation was in response to neutral videos. That is, in contrast to the aforementioned studies, emotion-independent coding is also impaired in PD. This degraded basic emotion-independent coding processing affects facial emotion processing as well [38–40]. These studies are consistent with our N250 findings. The fMRI is compatible with the delayed response N250 as its temporal resolution is lower than ERP.

In the current study, there was a correlation between the number of face-pareidolia responses and components of ERP in the bilateral frontal, temporal, and parietal cortices. In addition, we argue that posterior cortical dysfunction may play a critical role in face-pareidolia processing in PD. PD patients experience pareidolia, a complex visual illusion, more frequently than the controls. A dysfunction of the ventral visual pathway and disruption of the cholinergic projection to the frontotemporal cortex may be the other possible neural mechanisms. Our findings on face pareidolia support the results of researchers' study [19, 41], which demonstrated hypometabolism in the lateral occipital cortex and the temporoparietal cortex.

Like photo inversion of facial expression, pareidolia may cause disruption to face-specific configuration. If the pareidolia was processed like an altered face rather than an object, we would expect an amplitude increase and latency delay in the N170 wave as in inversion only. However, our findings showed that the prolonged latency but lower amplitude N170 potentials emerged with face-pareidolia stimulation. This was the case in both controls and Parkinson's patients. On the other hand, N170 amplitudes were larger and latencies were delayed for both face and face-pareidolia stimuli in patients with Parkinson's disease. This may indicate that processing;, whether face or face pareidolia, is slowing down in Parkinson's patients. Perhaps the object-sensitive areas are activated, whether face or face pareidolia. Our VPP findings were consistent with the N170 findings.

Although it has been shown that N250 ERP is triggered when famous faces are recognized, familiar faces cannot trigger this potential [42], and we have achieved this ERP potential, both, in patients with PD and in the control group. The amplitude of N250 was lower in Parkinson's patients. This may suggest that the activated neural network, which we assume may be enlarged in the temporoparietal response to face pareidolia in Parkinson's patients, is limited in the frontal region.

Evoked potentials indicate the real-time state of the physiological system. In order to carry out time-dependent physiological processes without interruption, the structure, neuromediators oscillation and response, and electrical impulse conduction should be normal. It was disrupted in patients with PD. It has been well-documented that vision, smell, hearing, and taste sensations are impaired in patients with PD [43–47]. Dopamine deficiency may have been a factor at the periphery, but not necessarily at the central transmission pathways. A component of delay in evoked potentials may also be due to peripheral dopamine deficiency.

Parkinson's disease has recently become noticeable with nonmotor symptoms, not motor findings. The human brain reacts to stimuli. The rate of reaction to stimuli depends on age, but it is also affected by diseases. Similar studies in neurodegenerative diseases such as Alzheimer's disease [48, 49] and schizophrenia [50] have also shown that these processes are interrupted. In this study, we examined the ERPs of early-stage Parkinson's patients with mild motor dysfunction in response to not only facial processing but also face-pareidolia processing, and we found that even in the early stages of PD these processing has deteriorated. These findings suggest that face and face pareidolia are continuous phenomena and might share underlying mechanisms.

Visual hallucination susceptibility in patients with PD is related to be consistent with a loss of cortical cholinergic input owing to changes in cholinergic associated with electrophysiological measures [51]. Increased delta responses over parietal and occipital locations during a facial expression emotional paradigm have been shown in Güntekin et al.'s study [52]. Three studies have reported ERP data on emotional faces in PD patients up to now. The first study reported an ERP study on neural generators revealed diminished amygdala responses N100 activity for fearful faces in PD patients [53]. The second study, which focused on P100 or N170 alterations, found impairment at later stages for emotion discrimination in PD patients [2]. The third study focused on dynamic facial expressions in PD patients and reported delayed and attenuated VPP component during the first 200 ms of processing dynamic faces [54].

Our study had some limitations. The lack of facial expressions during ERP recording was a limitation for the present study. The study was limited by the fact that the PD cohort consisted of PD patients in H and Y 1–2, and all PD patients were under dopamine replacement therapy, which might also affect the test performance. The neuropsychological functions of patients such as attention and visual-spatial memory were not explored beyond self-reports and our inquiry. We also did not examine the effect of dopamine treatment dose on ERPs. All this will be the subject of our future work.

## 5. Conclusion

Our study is the first that examined ERPs for both face and face pareidolia in patients with PD. The results of our study showed that ERPs associated with face processing is changed in early-stage Parkinson's patients. ERPs may even be neurobiological markers in the future. More longitudinal studies are needed.

## Figures and Tables

**Table 1:** Demographic and clinical characteristics of PD patients and healthy controls (HCs).

	PD patients *M* ± SD	HCs *M* ± SD
Number	21	26
Gender, M : F	15 : 6	14 : 12
Age, y	57.62 ± 8.04	56.22 ± 7.74
MMSE	25.1 ± 1.6	28.7 ± 3.1
Disease duration, y	2.2 ± 1.2	N/A
UPDRS	16.22 ± 3.54	N/A
Hoehn and Yahr stage	1.2 (1-2)	N/A

M: mean; SD: standard deviation; PD: Parkinson's disease; HCs: healthy controls; UPDRS: the Unified Parkinson's Disease Rating Scale (motor); MMSE : standardized Mini-Mental Test for General Cognitive Assessment; N/A: not applicable; M: male; F: female.

**Table 2:** Mean peak amplitude (in *μ*V ± SE) and latency (in ms ± SE) of N170, VPP, and N250 components.

Group	Condition	N170		VPP		N250	
		Latency	Amplitude	Latency	Amplitude	Latency	Amplitude
PD patients	Face	176.6 (1.83)	−4.21 (0.77)	173.9 (1.80)	2.39 (0.76)	223.5 (5.34)	−0.92 (0.48)
	Face pareidolia	180.5 (1.61)	−2.70 (0.68)	173.2 (1.6)	0.49 (0.47)	227.3 (3.3)	−1.83 (0.48)

HCs	Face	166.6 (0.95)	−1.21 (0.36)	169.0 (1.07)	−0.31 (0.33)	231.3 (2.17)	−3.76 (0.5)
	Face pareidolia	175.4 (1.3)	0.15 (0.37)	168.0 (1.07)	−0.41 (0.33)	229.2 (2.60)	−3.69 (0.52)

PD : Parkinson's disease; HCs : healthy controls; SE = standard error.

## Data Availability

The numeric data used to support the findings of this study are included in the article.
